# Supplementary material for: Factors influencing birth preparedness and complication readiness among childbearing age women in Thatta district, Sindh
Source: PLoS One. 2022 Sep 29;17(9):e0275243. doi: 10.1371/journal.pone.0275243 (PMC9522263; doi:10.1371/journal.pone.0275243)
Supplement: Attachment — Submitted filename: Response to Reviewers Comments.docx [file pone.0275243.s001.docx]

**Following are the answers of the raised points of academic editor and reviewers**.

1. **Kindly using recent literature explore the situation of birth preparedness and maternal health situation of Sindh province**

The recent literature of birth preparedness (Reference number 12, 19, 20) and maternal health situation of Sindh (Reference number 15, 17, 18) have added.

2. **Provide a flowchart to show the sample selection process**

The flow chart of multistage cluster sampling have added

3. **Mention whether you performed any normality tests.**

The normality test by mean median mode were performed and data was normally distributed

4. **Please review your reference list to ensure that it is complete and correct.**

All references have completed and corrected

**Specific Reviewers Response from the Authors**

***Reviewer #1:***

1. No major issue was identified. Rationale for conducting study is nicely explained.

**Ans: Thank you.**

2. Methodology- A mention of what constitutes a cluster unit, cluster size and number of clusters in multi-stage cluster sampling would have been better.

**Ans: Flow chart of cluster sampling have added for better understanding.**

3. Introduction and discussion can be made shorter & crisper by avoiding reputations of similar arguments.

**Ans: The reputations of similar argument have removed**

***Reviewer #2:***

1. The data and results could have been represented in a better way with all the values.

**Ans: The data and all the results have represented with all values**

2. There is no reference for the information stated in the sentence "Maternal deaths are more prevalent in developing countries due to lack of BPCR" in the introduction. Please add a reference.

**Ans: The reference number 10 have added**

3. No exact criteria is defined for the inclusion or the exclusion of the subjects in the paper. For the selection of the subjects,it's stated "multistage cluster sampling" was used in the methodology. However, why the exact numbers are not specified for each stage of cluster sampling?

**Ans: Those women who had delivered in the last 12 months were included in this study due to recall bias, and multistage cluster sampling were used which is further clearly defined by added flow chart of cluster sampling, and exact number of 65 women were added in the third stage of sampling by random sampling**

3. There is a typo in the sentence "The practice of birth preparedness and complication readiness in this study was found to be low i.e. 21.2%, as compared to other developing countries tudies such as Ethiopia (37%), India (58.5%) and in West Bengal" in Discussion. Wrong information: West Bengal is not a country, it's a state in the country, India. Please correct the typo and the information.

**Ans: The typo mistake and information has been corrected.**

4. Please correct the typo in the sentence "This variation might be due to socio-cultural and study settings differences as well as d implementation of related health programs".

**Ans: The typo mistake has been corrected.**

5. It will be good to add some recent reviews or references of BPCR (from2021) in the paper.

**Ans: Recent reviews or references of BPCR (2021) no: (12,19,22,23,24,28,31,35 and 36)**

***Reviewer #3:***

The article addresses one of the crucial issues faced by women in the society, especially in the developing world. The authors have used appropriate methodology and statistical analytical techniques to examine the issue. Some justification on sampling the women who gave birth to children in past 12 months would have enhanced the value of research.

**Ans: Due to recall bias those women who gave birth in past 12 months were included in this study, it is very difficult for women to recall previous complications related to pregnancy and delivery.**
