# Supplementary material for: Factors influencing birth preparedness and complication readiness among childbearing age women in Thatta district, Sindh
Source: PLoS One. 2022 Sep 29;17(9):e0275243. doi: 10.1371/journal.pone.0275243 (PMC9522263; doi:10.1371/journal.pone.0275243)
Supplement: Attachment — Submitted filename: Responses to reviewers plosone.docx [file pone.0275243.s002.docx]

**Factors influencing Birth preparedness and Complication Readiness among childbearing age women in Thatta district, Sindh**

***Responses to the editors' and reviewers' comments are in italics.***

**Editor/Reviewer Comments:**

*We thank and welcome the comments made by the editor and reviewers. We believe these comments will greatly improve this manuscript. Please see below for detailed responses to each comment. We have revised our manuscript as per their comments and suggestions.*

**Editors Comments**

**Comment 1:**

Missing details on the data collection procedures. Specifically, please report details of A) all the variables measured as part of the study B) how they were measured.

**Response:**

*We agree with the editor's suggestion. We have edited the methodology to include details of all the variables and how they were calculated (clean draft; Methodology, page 7, paragraph 2).*

**Comment 2:**

In addition, 3D effects in plots can bias and hinder interpretation of values, so avoid them in cases where regular plots are sufficient to display the data. We suggest replacing the plots in your manuscript with 2d versions.

**Response:**

*We agree with the editor's suggestion of changing the graphs to 2D. We have revised figures 2 and 3 on pages 10 and 13.*

**Revised Figure 2 (page 10)**

Choices for place of delivery

| Place of delivery | Perecentage |
| --- | --- |
| At home | 45.8% |
| BHU | 4% |
| DHQH | 13.9% |
| Private clinic | 16.9% |
| THQH | 7.1% |
| RHC | 12.2% |

**Revised Figure 3 (page 13)**

Score on six basic components of BPCR

| No of component | Percentage |
| --- | --- |
| 0 | 17.8% |
| 1 | 26.8% |
| 2 | 23.4% |
| 3 | 10.9% |
| ≥4 | 21.2% |
